# Special Issue on GNSS Data Processing and Navigation

**DOI:** 10.3390/s20154119

**Published:** 2020-07-24

**Authors:** Adria Rovira-Garcia, José Miguel Juan Zornoza

**Affiliations:** Research Group of Astronomy and Geomatics (gAGE), Department Physics, Universitat Politècnica de Catalunya (UPC), 08034 Barcelona, Spain; jose.miguel.juan@upc.edu

**Keywords:** high accuracy navigation, signal acquisition and tracking, orbit and clock determination, ionosphere, multipath, jamming, GNSS models, algorithms and techniques

## Abstract

Global Navigation Satellite System (GNSS) data can be used in a myriad of ways. The current number of applications exceed by far those originally GNSS was designed for. As an example, the present Special Issue on GNSS Data Processing and Navigation compiles 14 international contributions covering several aspects of GNSS research. This Editorial summarizes the whole special issue grouping the contributions under four different, but related topics.

## 1. GNSS Signals

The first stage in GNSS data processing involves acquiring the signals transmitted by the satellites. In this regard, ref. [1] proposed a tensor-based subspace tracking algorithm that mitigates multipath interference on receivers using multiple antennas, suitable for real-time applications. Sometimes, the interference can be intentional, ref. [2] evaluated the factors influencing the jamming on GNSS signals with the focus on high-end geodetic GNSS receivers. Finally, ref. [3] proposed a tracking loop able to perform such tracking of received signals in a highly accurate manner, which ultimately determine the accuracy of the positioning achievable. 

## 2. Atmospheric Modelling

The valuable data on the GNSS signals can be used to study the Earth derive a variety of models. One of the main propagation delays is originated at the upper atmosphere, precisely at the ionosphere. Ref. [4] studied the spatial and temporal variations of the Total Electron Content (TEC) at the Earth poles for one solar cycle.

## 3. High Accuracy Navigation

The accuracy of the computed coordinates by means of GNSS is enhanced when external information is received and combined with the GNSS measurements. Different strategies were presented to cope with outages occurring in the communication link receiving either the measurements from a reference station [5] or precise satellite orbits and clock corrections [6].

The GNSS measurements at the user are corrected using well-established models. Ref. [7] investigated the effect of using different Antenna Phase Centre (APC) correction models focusing on high-end geodetic receivers. Ref. [8] analyzed the variation of the Differential Code Biases (DCBs) occurring for the Beidou GNSS using a 40 m diameter, low-noise, and high-gain antenna.

Once the GNSS data is corrected, the user applies different algorithms to estimate accurately its Position Velocity and Time (PVT). In the case of receivers on the ground, ref. [9] assessed the efficiency of different filter strategies to perform such estimation, and ref. [10] addressed the variances of the different GNSS constellations to weigh them optimally. Finally, ref. [11] presented PVT based on the integration of GNSS measurements with other sensors, focused on autonomous vehicles.

GNSS can be used to determine the PVT of receivers on board of a satellite. Ref. [12] improved the determination of the orbit of the Gravity Recovery and Climate Experiment (GRACE) twin satellites through a modified clock estimating method. Furthermore, ref. [13] presented a positioning algorithm for space-borne GNSS timing receivers, assessing the Ling Qiao Low Earth Orbit (LEO) Chinese satellite.

In the inverse problem, knowledge of station coordinates can be used to determine the computation of satellite coordinates and associated clock biases, which is critical to the whole GNSS. In this regard, ref. [14] presented a characterization and evaluation of the atomic clocks onboard the Japanese system, namely the Quasi Zenith Satellite System (QZSS).
